# Role of Hyperglycemia in the Senescence of Mesenchymal Stem Cells

**DOI:** 10.3389/fcell.2021.665412

**Published:** 2021-04-15

**Authors:** Min Yin, Yan Zhang, Haibo Yu, Xia Li

**Affiliations:** Key Laboratory of Diabetes Immunology, Ministry of Education, Department of Metabolism and Endocrinology, National Clinical Research Center for Metabolic Diseases, The Second Xiangya Hospital of Central South University, Changsha, China

**Keywords:** mesenchymal stem cells, senescence, hyperglycemia, diabetes mettitus, mitochondrial dysfunction

## Abstract

The regenerative and immunomodulatory properties of mesenchymal stem cells (MSCs) have laid a sound foundation for their clinical application in various diseases. However, the clinical efficiency of MSC treatments varies depending on certain cell characteristics. Among these, the roles of cell aging or senescence cannot be excluded. Despite their stemness, evidence of senescence in MSCs has recently gained attention. Many factors may contribute to the senescence of MSCs, including MSC origin (biological niche), donor conditions (age, obesity, diseases, or unknown factors), and culture conditions *in vitro.* With the rapidly increasing prevalence of diabetes mellitus (DM) and gestational diabetes mellitus (GDM), the effects of hyperglycemia on the senescence of MSCs should be evaluated to improve the application of autologous MSCs. This review aims to present the available data on the senescence of MSCs, its relationship with hyperglycemia, and the strategies to suppress the senescence of MSCs in a hyperglycemic environment.

## Introduction

Mesodermal mesenchymal stem cells (MSCs) have self-renewal and multi-differentiation potential and can ultimately differentiate into osteoblasts, chondrocytes, and adipocytes (Timaner et al., 2020). The regenerative and immunomodulatory properties of MSCs have attracted the attention of researchers and doctors worldwide, because of their potential for cell-based therapy (Ankrum et al., 2014; Kornicka et al., 2018). Strong evidences exist to support the effectiveness of MSC treatments in managing degenerative diseases such as diabetes mellitus, myocardial infarction, liver failure, osteoarthritis, and Alzheimer’s disease (Hunsberger et al., 2016; Packer, 2018; Shi et al., 2018; Wang, 2018). However, the inconsistent results of many clinical trials have hampered their clinical application (Galipeau and Sensébé, 2018). Currently known reasons for these inconsistencies include MSC origin (biological niche), donor characteristics (age, obesity, diseases, or unknown factors), and culture conditions *in vitro* (Costa et al., 2020). However, the senescence of MSCs has often been overlooked. Autologous MSCs are considered auspicious because patient-derived cells are easily available and do not require sustained immunosuppression (Golpanian et al., 2016), but their senescence due to the personalized microenvironment should be addressed.

Hyperglycemia, oxidative stress, and altered immune reactions are prominent features of the diabetic microenvironment, all of which have been demonstrated to change bone marrow-derived MSC (BMSC) and adipose-derived MSC (AMSC) properties and functions, leading to MSC senescence (Cramer et al., 2010; Mahmoud et al., 2019). Moreover, umbilical cord-derived MSCs (UCMSCs) extracted from obese/diabetic mothers display premature senescence and mitochondrial dysfunction (Kim et al., 2015). With the rapidly increasing prevalence of diabetes and gestational diabetes mellitus, the effects of hyperglycemia on the senescence of MSCs should be addressed for a better application of autologous MSCs. In this review, we summarize published studies on the senescence of MSCs in terms of their definition and hallmarks. Moreover, we review how hyperglycemia drives MSC senescence. Finally, we recommend strategies to suppress MSC senescence in the diabetic microenvironment.

## Definition of MSC Senescence

The definition of senescence can be conceptually distinguished from that of aging, with that of senescence emphasizing the cellular level (Hayflick and Moorhead, 1961). Aging is the determining risk factor for most diseases and conditions that limit health span, and seven pillars promote aging, including metabolism, macromolecular damage, epigenetics, inflammation, adaptation to stress, proteostasis and stem cell regeneration. More importantly, these seven pillars are highly intertwined and their interplay plays a crucial role in the aging process (Kennedy et al., 2014). However, “cellular senescence is a cell state triggered by stressful insults and certain physiological processes, characterized by prolonged and generally irreversible cell-cycle arrest with secretory features, macromolecular damage, and altered metabolism” (Gorgoulis et al., 2019). Cellular senescence occurs in various physiological and pathological processes, such as embryogenesis, wound healing, injury, tumor suppression, and aging (Calcinotto et al., 2019). In addition, senescent cells are associated with aging-related diseases, such as type 2 diabetes, atherosclerosis, osteoporosis, glaucoma, and neurodegeneration (He and Sharpless, 2017). The exclusion of senescent cells or compounds that block the senescence-associated secretory phenotype (SASP) has been proposed for the treatment of cancer and aging-related diseases, which might play a major role in extending the health span (Partridge et al., 2020).

Interestingly, MSCs have also been found to be senescent *in vivo* and *in vitro* (Lin et al., 2019; Banimohamad-Shotorbani et al., 2020), which might lead to altered biological function and reduced therapeutic effect. Recently, we are seeing increased interest in MSC-based therapy for improving aging-related disorders and autoimmune diseases. Thus, the precise detection of senescent MSCs and elimination or reversion of the phenotype are essential for MSC-based therapy.

## Triggers and Hallmarks of MSC Senescence

MSC senescence can be induced by many intrinsic triggers and oncogenes, as well as physiological and pathological changes in their microenvironment. Based on the nature of the triggers, MSC senescence can be categorized into various types, including replicative senescence, stress-induced senescence, oncogene-induced senescence, and developmental senescence (Zhou et al., 2020). The hallmarks (phenotypic indicators) of MSC senescence vary with the nature of the causes that drive the different types of senescence.

The classic phenotype of cellular senescence include cell-cycle arrest, SASP, macromolecular damage, and deregulated metabolic profile (Gorgoulis et al., 2019). Cell-cycle withdrawal is the nature of cellular senescence. An overwhelming amount of studies have demonstrated that SASP (Ozcan et al., 2016) can reinforce senescence and affect other cells through an autocrine and paracrine mechanism (Acosta et al., 2008; Kuilman et al., 2010). Moreover, SASP can induce a local inflammation with compound effects. Both effects are essential for tissue or organ regeneration and reconstruction (Kuilman and Peeper, 2009). This secretome is mainly composed of various extracellular growth factors, including transforming growth factor beta (TGF-β), epidermal growth factor (EGF), platelet derived growth factor, hepatocyte growth factor, and insulin-like growth factor 1-binding proteins, in addition to cytokines/chemokines, receptor decoys, receptor antagonists, and extracellular matrix remodeling proteins (Freund et al., 2010). Among macromolecular damage and deregulated metabolic profile, DNA damage (telomere shortening), chromatin remodeling, autophagy, and senescence-associated epigenetic expression changes (Sengupta and Seto, 2004; Jung et al., 2010; Li et al., 2011; Franzen et al., 2017), mitochondrial dysfunction, and lysosome dysfunction play a major role in MSC senescence.

Compared with common cellular senescence, senescent MSCs have their own specific characteristics, including multipotentiality loss, cell phenotype changes, immunomodulatory property damage, and homing and migration damage (Campisi and d’Adda di Fagagna, 2007). Many studies have shown that the osteogenic differentiation potential of MSCs declines with age (Garcia-Sanchez et al., 2019; Zhou et al., 2019). However, there is no definite conclusion regarding adipogenic differentiation, although most of the studies have demonstrated that the adipogenic differentiation potential of MSCs tends to deteriorates with successive passages under standard expantion conditions (Sugihara et al., 2018). In terms of cell phenotypes, early-passage and late-passage senescent MSCs showed similar levels of identification markers (positive for the markers CD90, CD73, and CD105, negative for the markers CD45, CD34, CD14, CD11b, CD79α, CD19, and HLA-DR) proposed by the International Society for Cellular Therapy, suggesting that their assessment may be restricted only to basic MSC characterization (Yu et al., 2014). In contrast, a number of studies found increased CD295 and CD264 (Madsen et al., 2017) and decreased Stro-1 (Bakopoulou et al., 2017; Redondo et al., 2018), CD106 (vascular cell adhesion protein 1) (Lu et al., 2019) and CD146 (melanoma cell adhesion molecule, MCAM) (Jin et al., 2016) during prolonged culture, suggesting their ability to mark senescent MSCs (Laschober et al., 2009).

## Detection of Senescent MSCs

A multi-marker, three-step workflow is recommended for identifying senescent cells: “first, assessing senescence-associated beta-galactosidase (SA-β-gal) activity and/or lipofuscin accumulation (SBB or GL13 staining); second, co-staining with other markers frequently observed in (p16INK4A, p21WAF1/Cip1) or absent in (proliferation markers, lamin B1) senescent cells; and third, identifying factors anticipated to be altered in specific senescence contexts” (Gorgoulis et al., 2019). This workflow is applicable to all cell types. Regarding MSCs, analysis of various molecules and biological processes, including cell cycle arrest; DNA damage; and transcriptional, epigenetic, and metabolic changes, has been recommended for senescence detection. The characteristics of senescent MSCs include flattened and enlarged morphology (Kim et al., 2016; Iwasaki et al., 2019), decreased number of colony formation units (Ganguly et al., 2019), increased SA-β-gal activity (Gnani et al., 2019), altered gene and protein expression (p16, p21, p53), shortened or dysfunctional telomeres (Bonab et al., 2006; Guillot et al., 2007), increased 8-oxo-dG or γH2AZ expression (markers of DNA damage), microsatellite instability (marker of genomic instability and deficient DNA repair), and abnormal global methylation. However, specific and unequivocal markers are still lacking, and for reliability, the evaluation of MSC senescence should be based on the assessment of several markers *in vitro* (Neri and Borzí, 2020).

## Characteristics of Senescent MSCs in the Hyperglycemic Microenvironment

**FIGURE 1:**
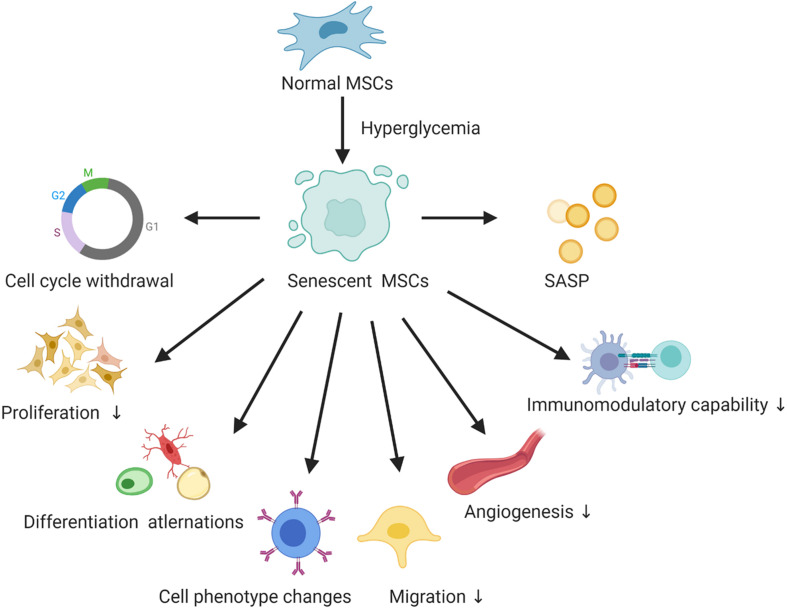
The hallmarks of senescent MSCs in the hyperglycemic microenvironment. SASP, senescence-associated secretory phenotype.

Type 2 diabetes mellitus (T2DM) is a chronic metabolic disorder that affects an increasing number of people worldwide (Zheng et al., 2018). Current treatment for diabetes failed to maintain long-term blood glucose homeostasis, resulting in insulin secretion deficiency and acute and chronic diabetic complications. Therefore, new approaches for the therapy of T2DM and its chronic complications are of special interest. Currently, cellular therapies involving autologous MSCs, especially those isolated from adipose tissue, constitute a promising treatment for diabetes (Bhansali et al., 2017). Offspring of women with GDM are at high risk of T2DM in adulthood, and MSCs derived from the umbilical cord and placental membrane offer a promising source of autologous MSCs for latent diabetes treatment. However, there are still many obstacles concerning the effectiveness of autologous MSCs isolated from diabetic patients and the effect of the microenvironment that need to be overcome before MSCs can be used for this purpose. A classic feature of senescent MSCs in the hyperglycemic microenvironment is cell-cycle arrest, as confirmed by senescence marker (p16, p53, p21) mRNA and protein expression (Liu J. et al., 2020). Additionally, increased SA-β-galactosidase staining is another important identification marker. Furthermore, senescent MSCs showed decreased proliferation, differentiation alternations, cell phenotype changes and declined migration, angiogenesis, and immunomodulatory capabilities. Though SASP plays a vital role in the common cellular senescence, only few studies have focused on the SASP of senescent MSCs in the hyperglycemic microenvironment, summarized in Figure 1.

### Proliferation

Proliferation is the most important characteristic of MSCs. Previous studies have shown that MSCs display lower proliferative capability and higher senescence in a hyperglycemic environment. The earliest study was conducted in 2010, when Cramer et al. showed that elevated glucose levels reduce the proliferative capacity of AMSCs with diabetes and AMSCs without diabetes, but the impact was more notable in AMSCs with diabetes. Similarly, AMSCs from patients with type 2 diabetes showed reduced viability and proliferative potential (Alicka et al., 2019). Intriguingly, incorporation of insulin enhances cell replication, especially in AMSCs without diabetes (Cramer et al., 2010). Another study demonstrated that diabetes mellitus was a significant determinant for forming a higher number of colony-forming units in BMSCs (Neef et al., 2012). Likewise, compared with human UCMSCs obtained from normal pregnant women, UCMSCs obtained from patients with GDM showed declined proliferative activity and increased senescence markers with a higher expression of p16 and p53 (Kim et al., 2015). In animal models, the colony formation capacity of rat adult non-adherent bone marrow MSCs (Na-BM-MSCs) was negatively influenced when cultured in high-glucose-containing medium; however, Na-BM-MSCs seemed to be relatively uninfluenced by aging, suggesting that diabetes, compared to aging, is more associated with reduced numbers and functional viability of MSCs *in vivo* (Stolzing et al., 2012). Taken together, these studies support the notion that hyperglycemia weaken the proliferation capacity of MSCs regardless of their sources both *in vitro* and *in vivo*. More importantly, hyperglycemia has a greater impact on MSC proliferation than does aging and this effect could not be completely restored by adding hypoglycemic agents.

### Differentiation

MSCs are characterized by their potent multipotency and do not only have mesodermal differentiation potential but also neurogenic differentiation potential. Current studies on the differentiation potential of senescent MSCs in hyperglycemia are controversial. It has been reported that the adipogenic and osteogenic differentiation capacities of AMSCs in high glucose-treated conditions were comparable to those in low glucose-treated conditions (Cheng et al., 2016). In contrast to this study, Cramer et al showed that that high glucose concentrations reduce the osteogenic and chondrogenic potential of AMSCs (Cramer et al., 2010). Likewise, UCMSCs from patients with GDM displayed significantly lower osteogenic and chondrogenic potential (Kong et al., 2019). Regarding the adipogenic potential of MSC under hyperglycemia conditions, there are some controversial results. Cramer et al. reported that high glucose concentrations enhanced the adipogenic potential of MSCs. Further, when adult human BMSCs were incubated with sera from T2DM patients for 14 days, the expression of adipogenic genes and Oil Red O staining were greatly increased (Moseley et al., 2018). However, GDM-UCMSCs demonstrated a significantly lower adipogenic potential (Kim et al., 2015; Moseley et al., 2018). Furthermore, another study found that BMSCs showed decreased adipogenic differentiation capability under high glucose conditions and that the mRNA expression of peroxisome proliferator activated receptor gamma, CCAAT/enhancer-binding protein alpha, leptin, and adiponectin in BMSCs decreased (Rharass and Lucas, 2019).

Interestingly, when cultured in an appropriate induction medium, both AMSCs from diabetic donors (dAMSCs) and from non-diabetic donors (nAMSCs) exhibited potential for trans-differentiation into neuron-like cells via a reactive oxygen species (ROS)-mediated mechanism (Cheng et al., 2016). Exposure to GDM may cause irreversible senescence and stress damage to endogenous amniotic MSCs; however, chorionic MSCs from GDM mothers can be efficiently reprogrammed into insulin-secreting cells and have a therapeutic potential comparable to that of chorionic MSCs from healthy mothers. Specifically, chorionic MSCs from both healthy and GDM mothers exhibited increased pancreatic transcription factor expression in parallel with retinoic acid, activin A, glucagon-like peptide-1, EGF, and other chemical components and could generate functional insulin-producing cells with betacellulin-sensitive insulin expression (Chen L. et al., 2020).

Overall, these data suggest that hyperglycemia plays a pivotal role in the differentiation process of MSCs, and this process depends on the different sources of MSC. In general, hyperglycemia weakens the osteogenic and chondrogenic differentiation capability, but interestingly, enhances trans-differentiation into neuron-like cells and insulin-secreting cells. However, there are no uncontested results regarding the adipogenic differentiation. This inconsistency may be due to the age of MSC donors, different passages of MSCs, and the extent and duration of hyperglycemia.

### Cell Markers

Markers for identification and stemness are major areas of interest within the field of MSC senescence under hyperglycemic conditions. It has been reported that the expression of cell surface markers in dAMSCs and nAMSCs was similar to that of MSCs (Cheng et al., 2016). Moreover, senescent human UCMSCs, also express comparable levels of CD73, CD90, CD105, and marker proteins (Kim et al., 2015). Additionally, when cultured in high and normal glucose conditions, MSCs in the endosteal niche lining compact bone were not altered, demonstrating the presence of analogous MSC (CD73/CD105/CD90), embryonic (Slug and Snail), and multipotency (CD146) markers throughout the extended culture (Al-Qarakhli et al., 2019).

However, MSCs under hyperglycemia showed negligible pluripotency (Nanog, Oct4) and hematopoietic (CD34/CD45) markers (Al-Qarakhli et al., 2019). Recently, one study showed that stemness markers were significantly lower in insulin-controlled GDM mothers-UCMSCs and diet-controlled GDM mothers-UCMSCs than in normal UCMSCs (Kong et al., 2019). Another study using a systems biology approach identified widespread downregulation of MSC markers in the subcutaneous adipose tissue of diabetic rats (Ferrer-Lorente et al., 2014). Likewise, nucleus pulpous-derived MSCs cultured with high glucose showed decreased expression of stemness genes as well as decreased mRNA and protein expression of silent information regulator protein 1 (SIRT1), SIRT6, glucose transporter 1, and hypoxia inducible factor-1α (HIF-1α), but enhanced cell senescence, cell apoptosis, and caspase-3 expression (Liu Y. et al., 2020). Contrary to these data, Cheng et al. reported that dAMSCs or high-glucose treated nAMSCs presented higher expression of the pluripotent markers Sox-2, Oct-4, and Nanog, and this effect was related to ROS-mediated Akt attenuation. With antioxidant treatment, high glucose-treated nAMSCs exhibited enhanced cell growth but no stemness enhancement (Cheng et al., 2016). This rather contradictory result may be due to individual difference in MSC donors. Collectively, these studies indicate that hyperglycemia has a little effect on the identification markers, but decreases the stemness of MSCs in most cases.

### Other (SASP, Immunomodulatory Properties, Angiogenesis, Migration, Insulin Resistance)

In addition to the above features, MSCs in hyperglycemic conditions show decreased immunomodulatory properties (Montanucci et al., 2016), angiogenesis (Xiao et al., 2020), and migration (Liu Y. et al., 2020), but increased SASP and insulin resistance. SASP is mainly composed of various extracellular growth factors, cytokines/chemokines and receptor antagonists. It is well known that the proliferation and cytokine secretion (TGF-β and VEGF) of MSCs are inhibited after long-term culture even in MSCs derived from young donors (Baek et al., 2018). One study showed that aging and metabolic changes in diabetes also modified the MSC cytokine production abilities (Kondo et al., 2015). Moreover, another study showed that AMSCs from T2DM patients exhibited lower adiponectin, VEGF, and chemokine ligand-12 secretion, but demonstrated an overproduction of leptin (Alicka et al., 2019). Interestingly, data also showed that a high glucose environment causes prominent disparities in the expression of genes involved in insulin resistance, such as resistin and adiponectin between nAMSCs and dAMSCs. Some changes in the expression of these genes were permanent in dAMSCs when treated with insulin (Cramer et al., 2010). Overall, the evidence presented in this section suggests hyperglycemia adversely affect the biological function of MSC which is critical for their therapeutic effect.

## Mechanisms of Senescent MSCs in the Hyperglycemic Microenvironment

**FIGURE 2:**
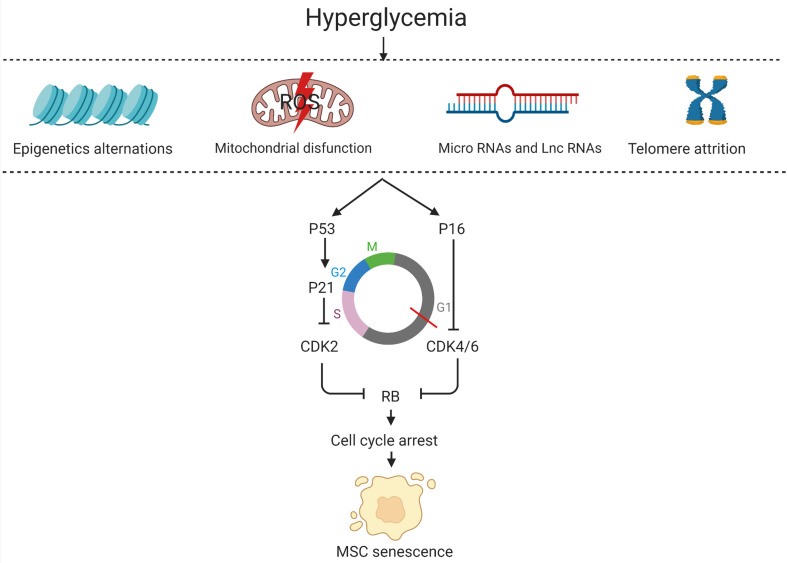
The mechanisms of senescent MSCs in the hyperglycemic microenvironment.

Cell-cycle withdrawal is the core feature of MSC senescence. High glucose results in a significant decline in the number of cells in the G0/G1 phases and an increase in cells in the S and G2/M phases, suggesting that high glucose induces cell cycle withdrawal of MSCs. Besides, cell cycle markers (p16, p21, p27, and p53) were surprisingly upregulated in insulin-controlled GDM mothers-UCMSCs and diet-controlled GDM mothers-UCMSCs compared to normal UCMSCs (Kong et al., 2019). The increase in the expression of all these cell cycle markers results in increased levels of cyclin-dependent kinase inhibitors (CKD) which actuate entry into senescence by triggering Rb to block cell cycle progression, summarized in Figure 2. Apart from this classical pathway, studies have shown that phosphorylation of p38 mitogen-activated protein kinase (MAPK) in MSCs was increased under high glucose conditions without alterations of total p38 MAPK levels. The study also revealed that the p38 MAPK inhibitor SB203580 can alleviate the phosphorylation of p38 MAPK (Qi et al., 2019). In the next sections, brief discussions have been presented on the mechanisms of senescent MSCs under hyperglycemic conditions from the aspects of mitochondria dysfunction, microRNAs, telomerase and epigenetics, summarized in Figure 2.

### Mitochondrial Dysfunction and Oxidative Stress

Mitochondrial dysfunction is a vital factor during MSC senescence in the hyperglycemic microenvironment and has been extensively investigated. A previous study showed that GDM-UCMSCs displayed significantly declined mitochondrial activity and low expression of the mitochondrial function regulatory genes COX1, PGC-1a, ND2, ND9, and TFAM (Kim et al., 2015). Similarly, another study demonstrated that the gene expression of antioxidant enzymes and that of mitochondrial function genes (NADH dehydrogenase subunit 2, mitochondrial transcription factor A (TFAM), peroxisome proliferator-activated receptor γ coactivator-1alpha, and NADH dehydrogenase (ubiquinone) 1β-subcomplex) were obviously lower in UCMSCs from GDM mothers than in UCMSCs from healthy mothers (Kong et al., 2019). Additionally, AMSCs from type 2 diabetes patients exhibit a senescence phenotype and mitochondrial dysfunction because of excessive oxidative stress (Alicka et al., 2019). Moreover, high glucose-treated nASCs exhibited declined cell migration and higher intracellular ROS than did low glucose-treated cells. This high glucose-induced biological reaction is associated with ROS-mediated Akt attenuation (Cheng et al., 2016). Consistent with previous studies, Rharass et al. showed that intracellular and extracellular ROS concentrations were significantly increased by 50% under high glucose exposure, paralleled by increased mRNA levels of the H_2_O_2_ generating enzyme NADPH oxidase 4 (Rharass and Lucas, 2019). All of the studies reviewed here support the notion that hyperglycemia drive mitochondrial dysfunction through the ROS pathway which is fundamental to the senescence of MSCs.

### MicroRNAs and lncRNAs

MicroRNAs (miRNAs) and lncRNAs are small non-coding RNAs that regulate gene expression, playing a pivotal role in diverse biological processes, including MSC senescence. One study showed that AMSCs isolated from diabetes patients or cultured in a medium containing glycation end products (AGEs) showed decreased stem cell activity, differentiation potential, and angiogenesis effect. Moreover, the expression of miRNA-1248 was reduced, paralleled by increased expression of CBP/p300-interacting transactivator with ED-rich tail 2 (CITED2), an inhibitor of HIF-1α, which influences growth factors that promote cellular proliferation, angiogenesis, and wound healing. Thus, the authors of the study concluded that glucolipotoxicity impaired the effectiveness of AMSCs through the miR-1248/CITED2/HIF-1α pathway (Xiao et al., 2020). As previously mentioned, several studies have shown that hyperglycemia reduces the osteogenic potential of MSCs. Recently, one study explored the core molecular mechanism and found that stearoyl-coenzyme A desaturase-1 (SCD1) expression was downregulated in T2DM patients, and decreased SCD1 reduced the osteogenic differentiation of BMSCs by activating the miR-203a/FOS and miR-1908/EXO1 regulatory pathways (Chen Y.S. et al., 2020). In addition, a previous study found obvious differences in the expression of miRNAs involved in cell proliferation (miR-146a-5p, miR-16-5p, and miR-145-5p), together with miRNA and genes responsible for insulin sensitivity and glucose homeostasis (miR-17-5p, miR-24-3p, 140-3p, SIRT1, TGFβ, HIF-1α, LIN28, and FOXO1). More importantly, the authors observed a similar correspondence between miR-16-5p, miR-17-5p, miR-24-3p, 140-3p, miR-145-5p, and miR-146a-5p expression in the extracellular vesicle fraction. Apart from miRNAs, lncRNAs are also involved in the MSC senescence triggered by hyperglycemia, and it has been shown that high glucose inhibits osteogenic differentiation of MSCs through the lncRNA AK028326/CXCL13 pathway, revealing new molecular mechanism of many osteogenesis-related diseases, especially in patients with diabetes mellitus (Cao et al., 2016).

### Telomerase and Telomere Length

The telomere is a region of repetitive nucleotide sequences at each end of a chromatid, which protects the end of the chromosome from deterioration. Telomerase attrition is one of the hallmarks of cellular senescence. One study showed that telomerase was significantly lower in UCMSCs from GDM mothers than in UCMSCs from normal mothers (Kong et al., 2019). Moreover, the expression of the positive telomere maintenance marker (rTERT, TR) in MSCs in the endosteal niche lining compact bone was downregulated under high glucose conditions. Interestingly, telomere length is altered throughout *in vitro* expansion, with hyperglycemia markedly decreasing telomere lengths at PD50 and PD200 (Al-Qarakhli et al., 2019). Together, these studies recognize telomere attrition as one of the mechanisms of senescent MSCs in the hyperglycemic microenvironment.

### Epigenetics

Epigenetics is one of the pillars of aging and MSC senescence which can be detected by epigenetic modifications. One study showed that cardiac mesenchymal cells from patients with T2DM were characterized by premature cellular senescence, reduced proliferation, decreased differentiation potential, and decreased phosphorylation at histone H3 serine 10. Global histone code profiling of cardiac MSCs rom patients with T2DM demonstrated that acetylation of histone H3 lysine 9 (H3K9Ac) and lysine 14 (H3K14Ac) was reduced, while the trimethylation of H3K9Ac and lysine 27 was greatly increased. Moreover, DNA CpG island hypermethylation was detected at the promoter of genes involved in genomic stability and cell growth control. These results reveal that epigenetic changes may be an essential factor in MSC senescence in hyperglycemia (Vecellio et al., 2014).

### Other

MSCs from streptozotocin-induced diabetic rats displayed decreased proliferation and osteogenic differentiation potential, but increased senescence and apoptosis, and these effects appeared to be mediated by increased AGEs and an jincrease in the receptor for AGEs (Stolzing et al., 2010). Diabetic BMSCs showed inhibited osteogenesis and muscle ARNT-like protein 1 (BMAL1) expression, and over-expression of BMAL1 could recover BMSC osteogenesis in T2DM partly by reducing GSK-3β expression to trigger the Wnt/β-catenin pathway (Li et al., 2017).

## Strategies to Suppress Senescence of MSCs in the Hyperglycemic Microenvironment

**FIGURE 3:**
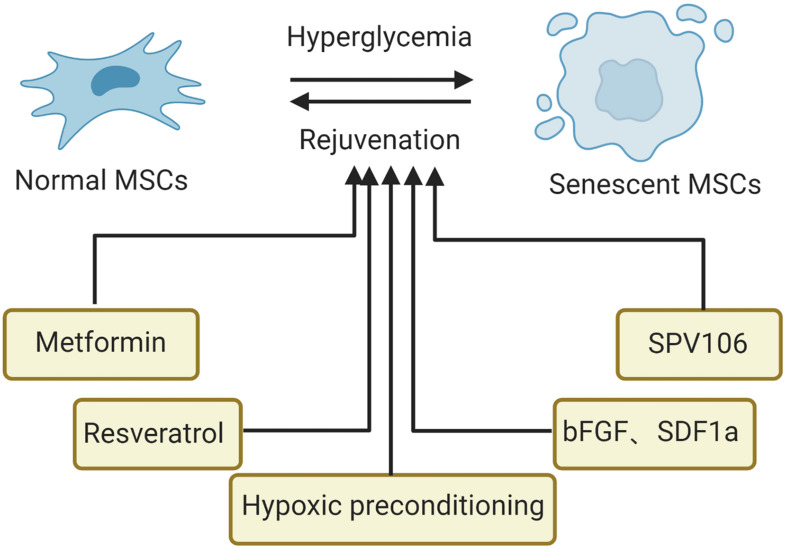
Strategies to suppress senescence of MSCs in the hyperglycemic microenvironment. SCD1, stearoyl-coenzyme A desaturase-1; bFGF, basic fibroblast growth factor; SPV106, histone acetylase activator pentadecylidenemalonate 1b.

Cellular senescence has been proven to be reversible. At present, the most promising drugs for this purpose include rapamycin, senolytics, and metformin (Partridge et al., 2020). For senescent MSCs, in addition to drugs, genetic modification, microRNA treatment and preconditioning modification are important approaches for rejuvenation (Neves et al., 2017; Ocansey et al., 2020). Currently, strategies to block the senescence of MSCs in hyperglycemic conditions include drugs (metformin, resveratrol, cytokines, and histone acetylase activator) and preconditioning modification (hypoxia), summarized in Figure 3. Besides, various *in vitro* experiments demonstrated that good glucose control could alleviate the senescence of MSCs, suggesting that blood glucose control should be advised to prevent or limit senescence of MSCs in patients with diabetes. More importantly, for MSCs from normal or diabetic donors, the appropriate glucose control in the MSC expansion process and in the transplantation process is essential for the clinical efficiency of MSC treatments (Lo et al., 2011).

Metformin is widely prescribed for T2DM; interestingly, recent studies have suggested an important role for metformin in mitigating aging. After treatment with metformin, AMSCs isolated from mice showed reduced cellular senescence and decreased ROS but increased proliferative potential and osteogenic differentiation potential and declined adipogenic differentiation (Marycz et al., 2016). Resveratrol, a small polyphenol, has emerged as a potential therapy due to its anticancer and anti-inflammatory properties. In diabetic animal models, AMSCs preconditioned with resveratrol showed increased stem cell function and better effects (Chen et al., 2019). One of the mechanisms is that resveratrol increases the expression of the survival marker p-Akt, resulting in enhanced AMSC viability (Cheng et al., 2016). It is well known that the proliferation and cytokine secretion (TGF-β and VEGF) of MSCs are inhibited after long-term culture even in MSCs when derived from young donors (Baek et al., 2018). However, this situation could be changed by the addition of substance P, suggesting that substance P could block the loss of the therapeutic potential of MSCs by preserving their proliferative and paracrine potential. Moreover, another study found that basic fibroblast growth factor promotes the proliferation and inhibits the apoptosis of AMSCs isolated from patients with T2DM by reducing cellular oxidative stress (Nawrocka et al., 2017). Similarly, when pretreated with a mixture of Stromal cell-derived factor-1 alpha and bFGF, insulin-producing cells differentiated from AMSCs showed maximally alleviated senescence, apoptosis, and cell damage. Interestingly, these AMSCs demonstrated enhanced release of insulin, increased cell proliferation and upregulation of insulin 1, insulin 2, Ngn3, Nkx6.2, and Pdx1when triggered by hyperglycemia. Epigenetics, as one of the pillars of MSC senescence, has been studied for searching targets to suppress the senescence of MSCs. For example, the histone acetylase activator pentadecylidenemalonate 1b (SPV106) has been shown to alleviate cellular senescence and recover the regenerative and multi-differentiation potential of cardiac MSCs by restoring normal amounts of H3K9Ac and H3K14Ac and reducing DNA CpG hypermethylation (Vecellio et al., 2014). In addition, hypoxic preconditioning was able to enhance the regeneration potential of aging bone marrow MSCs into pancreaticβ-cells in streptozotocin-induced type-1 diabetic mice by altering gene expression levels of certain growth factors (Waseem et al., 2016). The underlying molecular mechanism might be related to hypoxia, which induces different regulation patterns in many cell cycle checkpoint genes such as HIF-1a, ataxia telangiectasia mutated, and ataxia telangiectasia and Rad3 related p53, p21, p27, and p21 (Sharma and Bhonde, 2015).

## Conclusion

MSCs are promising cells for the treatment of regenerative and immunomodulatory diseases. Various studies have shown that hyperglycemia may induce MSC senescence and diminish crucial functions of MSCs, such as cell proliferation, differentiation capacity, angiogenesis and immunomodulatory capability, which remarkably restrict their therapeutic efficiency. The appropriate use of MSCs for clinical applications demands a general knowledge of the MSC senescence process. Additionally, approaches that generate large populations of MSCs *in vitro* without affecting their regenerative or immunomodulatory properties need to be established. In this review we discussed the relevant literature to date and suggested possible methods to improve therapeutic efficacy through regulating specific factors or the hyperglycemia microenvironment associated with MSC senescence.

Unlike BMSCs and AMSCs, UCMSCs have a painless collection process, faster self-renewal properties and lower immunogenicity. UCMSCs are attractive autologous or allogenic agents for the treatment of cancer and aging-related diseases (Ding et al., 2015). However, gestational diabetes is progressively prevalent and predicted to influence more than 20 million livebirths (about one in six) worldwide (Saravanan, 2020). Thus, considerably more work will need to be done to investigate the senescence of UCMSCs in the hyperglycemic environment. Besides, uniform experimental studies to elucidate the molecular basis and signaling pathways regulating senescence in MSCs in a diabetic microenvironment are urgently required, especially in the field of mitochondrial dysfunction and epigenetics. These mechanistic studies would contribute to discovering new strategy to suppress MSCs senescence in the hyperglycemic microenvironment. Furthermore, studies involving the detection of the senescence of MSCs in DM-related chronic complications such as diabetic nephropathy, diabetic neuropathy, and diabetic retinopathy will be beneficial. New specific assays to identify senescent MSCs to predict the safety and potency of autologous MSCs in DM or GDM and new strategies to suppress the senescence of MSCs should be developed. Data from these studies would facilitate accurate evaluation of the efficacy of the treatment and provide more precise inclusion and exclusion criteria for the proper selection of patient groups with diabetes that would best benefit from the treatment.

## Author Contributions

MY searched literature and drafted the manuscript. YZ and HY searched literature. XL provided ideas and revised the manuscript. All authors read and approved the final manuscript.

## Conflict of Interest

The authors declare that the research was conducted in the absence of any commercial or financial relationships that could be construed as a potential conflict of interest.
